# Associations between long-term aircraft noise exposure, cardiovascular disease, and mortality in US cohorts of female nurses

**DOI:** 10.1097/EE9.0000000000000259

**Published:** 2023-06-21

**Authors:** Stephanie T. Grady, Jaime E. Hart, Francine Laden, Charlotte Roscoe, Daniel D. Nguyen, Elizabeth J. Nelson, Matthew Bozigar, Trang VoPham, JoAnn E. Manson, Jennifer Weuve, Sara D. Adar, John P. Forman, Kathryn Rexrode, Jonathan I. Levy, Junenette L. Peters

**Affiliations:** a Department of Environmental Health, Boston University School of Public Health, Boston, Massachusetts; b Channing Division of Network Medicine, Department of Medicine, Brigham and Women’s Hospital and Harvard Medical School, Boston, Massachusetts; c Exposure, Epidemiology and Risk Program, Department of Environmental Health, Harvard T.H. Chan School of Public Health, Boston, Massachusetts; d Department of Epidemiology, Harvard T.H. Chan School of Public Health, Boston, Massachusetts; e Division of Population Sciences, Dana-Farber Cancer Institute, Boston, Massachusetts; f College of Arts and Sciences, Boston University, Boston, Massachusetts; g Epidemiology Program, Division of Public Health Sciences, Fred Hutchinson Cancer Center, Seattle, Washington; h Division of Preventive Medicine, Brigham and Women’s Hospital and Harvard Medical School, Boston, Massachusetts; i Department of Epidemiology, Boston University School of Public Health, Boston, Massachusetts; j Department of Epidemiology, University of Michigan School of Public Health, Ann Arbor, Michigan; k Division of Women’s Health, Department of Medicine, Brigham and Women’s Hospital and Harvard Medical School, Boston, Massachusetts

**Keywords:** Aircraft, Transportation, Noise, Cardiovascular disease, All-cause mortality

## Abstract

There is limited research examining aircraft noise and cardiovascular disease (CVD) risk. The objective of this study was to investigate associations of aircraft noise with CVD among two US cohorts, the Nurses’ Health Study (NHS) and Nurses’ Health Study II (NHSII).

**Methods:**

Between 1994 and 2014, we followed 57,306 NHS and 60,058 NHSII participants surrounding 90 airports. Aircraft noise was modeled above 44 A-weighted decibels (dB(A)) and linked to geocoded addresses. Based on exposure distributions, we dichotomized exposures at 50 dB(A) and tested sensitivity of this cut-point by analyzing aircraft noise as categories (*<*45, 45–49, 50–54, ≥55) and continuously. We fit cohort-specific Cox proportional hazards models to estimate relationships between time-varying day-night average sound level (DNL) and CVD incidence and CVD and all-cause mortality, adjusting for fixed and time-varying individual- and area-level covariates. Results were pooled using random effects meta-analysis.

**Results:**

Over 20 years of follow-up, there were 4529 CVD cases and 14,930 deaths. Approximately 7% (n = 317) of CVD cases were exposed to DNL ≥50 dB(A). In pooled analyses comparing ≥50 with <50 dB(A), the adjusted hazard ratio for CVD incidence was 1.00 (95% confidence interval: 0.89, 1.12). The corresponding adjusted hazard ratio for all-cause mortality was 1.02 (95% confidence interval: 0.96, 1.09). Patterns were similar for CVD mortality in NHS yet underpowered.

**Conclusions:**

Among participants in the NHS and NHSII prospective cohorts who generally experience low exposure to aircraft noise, we did not find adverse associations of aircraft noise with CVD incidence, CVD mortality, or all-cause mortality.

**What this study adds**

While several studies in other countries have evaluated cardiovascular and mortality risks of aircraft noise exposure, this study is among the first to report on associations within the United States. This study takes advantage of large, national companion cohorts and multi-year, multi-airport aircraft noise assessments to explore these relationships. We did not find associations of aircraft noise with cardiovascular disease incidence or all-cause mortality, but note that even among these larger cohorts, we were limited by small numbers of exposed cases. This study highlights needs for future studies specifically designed to study noise exposure to capture exposure gradients and ascertain associations.

## Introduction

Noise, defined as unwanted or harmful sound, is a ubiquitous yet often overlooked environmental pollutant. The World Health Organization (WHO) estimates that in Western Europe alone, about 1.6 million disability-adjusted life-years are annually lost due to environmental noise through increased annoyance, sleep disturbance, and cardiometabolic diseases.^1^ Several studies have examined the impacts of road traffic noise exposure on cardiovascular health^2–14^; however, less is known regarding the potential impacts of aircraft noise, particularly in the United States.

Cardiovascular disease (CVD) is responsible for over 800,000 deaths in the United States^15,16^ and 17.9 million deaths worldwide^17^ each year; therefore, it is critical to examine the possible role of modifiable environmental factors, such as noise. Yet, limited research has examined associations between aircraft noise exposure and CVD.^3,5,6,18–26^ Within the United States, only a few studies have been conducted, in which authors found relationships between aircraft noise exposure and CVD-related events, yet were limited to zip-code-level data^18^ or one time point of exposure data.^24–26^ Furthermore, previous literature has shown mixed results when examining relationships between aircraft noise and CVD incidence, CVD mortality, and all-cause mortality.^3,5,6,18–23^ In a longitudinal study examining modeled annual average exposures to aircraft noise in Switzerland, the authors found positive associations between noise and mortality from myocardial infarction (MI) comparing ≥60 dB(A) with <45 dB(A), but not with all-cause or other cardiovascular-specific mortality.^19^ In an updated study from Vienneau et al on the same cohort with extended years from 2000 to 2011, investigators reported associations of noise with mortality from CVD and CVD-subtype mortality.^27^ Similar to its predecessor, the study did not find associations between aircraft noise and CVD mortality; however, it did find positive exposure–response associations with mortality from CVD subtypes.^27^

The aim of this study is to examine associations between long-term aircraft noise exposure and CVD incidence as well as CVD and all-cause mortality among two prospective cohorts in the United States with comprehensively determined exposure, outcomes, and individual- and area-level covariates.

## Methods

### Study populations

The Nurses’ Health Study (NHS) and Nurses’ Health Study II (NHSII) are two prospective cohorts of 121,700 and 116,429 female nurses followed since 1976 and 1989, respectively. Participants were recruited into NHS if they were female nurses born between 1921 and 1946 who were living in one of the 11 US states (California, Connecticut, Florida, Maryland, Massachusetts, Michigan, New Jersey, New York, Ohio, Pennsylvania, and Texas). Participants were recruited into NHSII if they were female nurses born between 1946 and 1964 who were living in one of 14 states (California, Connecticut, Indiana, Iowa, Kentucky, Massachusetts, Michigan, Missouri, New York, North Carolina, Ohio, Pennsylvania, South Carolina, and Texas). There are currently individuals in NHS and NHSII living in all 50 states and the District of Columbia. Questionnaires have been mailed every 2 years to participants asking about demographics, health status, lifestyle information, and family history. Additionally, detailed information on residential address history has been collected on each participant, and addresses have been geocoded at each time point. The response rate has been ~90%.^28,29^ The Institutional Review Board at Brigham and Women’s Hospital approved this study, and participants provided implied consent through return of questionnaires.

Follow-up for the current analyses occurred from 1994 through 2014 for NHS and from 1995 through 2013 for NHSII, given the availability of complete exposure and outcome data. We restricted the sample to individuals living close to one of the 90 airports studied to reduce potential confounding and exposure misclassification from residing near airports not included in this study. To define people living in close or similar geographic areas, we used a 22.2-mile (35.7 km) radius buffer around each of the 90 study airports, as this buffer size represents the maximal extent of aircraft noise assessment for these airports.^30^ For our CVD incidence analyses, but not mortality, we excluded participants with CVD (defined as MI or stroke) at or before baseline.

### Exposure assessment

Our exposure of interest, day-night average sound level (DNL), is the primary metric used in the US aviation decision-making. DNL represents an annualized 24-hour weighted average of noise that includes a 10 A-weighted decibel (dB(A)) penalty for nighttime noise. We obtained DNL contours from the John A. Volpe National Transportation Systems Center for 90 selected US airports in 5-year increments from 1995 to 2015 modeled using the Federal Aviation Administration (FAA)’s Aviation Environmental Design Tool.^31^ Methods for aircraft noise modeling have been published elsewhere,^32,33^ but briefly, the Aviation Environmental Design Tool utilized annualized flight track information, aircraft type, and time of day to estimate annual DNL at a 1 dB resolution down to 45 dB(A) at a 0.1 nautical mile (~607 feet) spatial resolution.

DNL contours were overlaid with participant geocoded addresses. For years between the five-year noise intervals, we assumed that levels at each airport did not change (applied noise estimate from the earlier year until new estimate was available). For participants living within noise contours from multiple airports, we calculated the combined noise exposure using the following formula: $CombinedLevel=10Log_{10}\underset{i=1}{\overset{n}{\sum }}\;(10^{\left(Li/10\right)})$, where *Li* represents the decibel level for the *i*th estimate.^32^ Additionally, we assigned participants who did not live within contours of the 90 airports DNL exposures of 44 dB(A) (below the minimum estimated noise level of 45 dB(A)).

### Outcome assessment

We assessed incident CVD as our primary outcome of interest, with all-cause and cardiovascular-specific mortality as secondary outcomes. At each biennial questionnaire period, participants reported if they had a CVD event, which were subsequently confirmed by medical records.^34^ Deaths were reported by next-of-kin, post office return notices, and searches of the National Death Index.^35,36^ The validity of the National Death Index was high, with two validation studies showing sensitivity over 96% and a specificity of 100%.^35,36^

We defined incident CVD as the first occurrence of nonfatal MI (ICD code 410), fatal coronary heart disease (CHD, ICD code 410 or 412), or nonfatal or fatal stroke (ICD codes 430-437). Information on case definitions in the NHS has been previously published.^34,37,38^ Briefly, events were adjudicated by physician review of medical records and were included in our analyses if they were classified as definite or probable. Definite MI and CHD cases were defined as having medical records or autopsy reports confirming an event. Additionally, definite nonfatal MIs met the WHO criteria of having typical MI symptoms and either elevated enzymes or diagnostic electrocardiographic findings.^34,37–39^ Probable MI and CHD cases were defined as having corroborated detailed information by participants through additional interviews or evidence of hospitalization or death certificate yet no medical records.^34^ Of note, we did not rely on death certificate coding alone for cases of CHD death. Definite stroke cases were classified according to the National Survey of Stroke criteria, requiring medical record documentation of a sudden onset of neurological symptoms lasting more than 24 hours or until death attributable to a cerebrovascular event.^34,40^ Cases of stroke that did not meet these criteria or had no medical records were considered probable.^34^ Probable CVD cases were included because analyses based on combined definite and probable cases yielded results similar to those based on definite cases alone.

We defined all-cause (nonaccidental) mortality using physician-classified and adjudicated primary cause of death from International Classification of Diseases, Eighth Revision (ICD-8) codes on death certificates and medical records.^38,41^ We included accidental causes of death in sensitivity analyses, given the potential pathway of aircraft noise to mortality through sleep impairment and associated increased accident risk. CVD mortality was further identified using ICD-8 codes for cardiovascular deaths (390.0 to 458.9 and 795.0 to 795.9).^42,43^

### Covariates

We selected covariates for our statistical models *a priori* based on literature review of potential predictors of noise and risk factors for CVD. We used fixed and time-varying self-reported information from the biennial questionnaires on demographics and medical history (race/ethnicity, menopausal status, family history of MI), individual-level socioeconomic status (marital status and spouse’s educational attainment), and health-related behaviors (smoking status, smoking history in pack-years, physical activity, and dietary intake). Physical activity was operationalized as the total energy expenditure in metabolic equivalent of task-hours per week.^38,44^ Diet and alcohol intake were assessed every 4 years using a food frequency questionnaire. Food frequency responses were also used to calculate the Alternate Healthy Eating Index, a validated diet index developed to predict the risk of CVD outcomes within the cohorts.^38,45^ Larger values represented closer adherence to a better-quality diet.^45^

We also accounted for time-varying neighborhood-level characteristics, such as neighborhood socioeconomic status, population density (persons per km^2^), and region of residence (Northeast, Midwest, South, and West) using data from the US Census for the corresponding (or closest to corresponding) year of study. Neighborhood socioeconomic status (nSES) was assessed by creating an index in which nine variables related to income, wealth, educational attainment, employment, and racial composition of the residential Census tract were standardized (z-score) and summed, with higher nSES index values indicating greater affluence.^46,47^ We utilized monthly fine particulate matter (PM_2.5_) data predicted at each address from a validated spatiotemporal model, as described previously.^48,49^ Briefly, the model used generalized additive mixed models to develop predictions for monthly PM_2.5_ concentrations using data from the US Environmental Protection Agency’s Air Quality System, several other air quality monitoring networks, and studies collecting PM_2.5_ measurements.^48,49^ For this analysis, monthly PM_2.5_ estimates were averaged into 2-year estimates, updated for each 2-year period, and modeled as a continuous variable.

### Statistical analyses

To estimate associations between noise and incident CVD and mortality, we used Cox proportional hazards models stratified by age and 2-year time period and calculated hazard ratios (HRs) and 95% confidence intervals (CIs). We calculated survival time in person-months of follow-up, from June 1, 1994 (NHS) or 1995 (NHSII) to the date of first CVD event, death, or May 31, 2014 (NHS) or 2013 (NHSII), whichever date came first. In mortality analyses, follow-up ended at date of death or 31 May 2014 (NHS) or 2013 (NHSII), whichever date came first. We tested for violations of the proportional hazards assumption by fitting Cox proportional hazards models with multiplicative interaction terms for noise and calendar time and additionally conducted likelihood ratio tests.

Although health-related behaviors and area-level exposures could potentially be mediators or modifiers of our primary association, it was possible that they could also confound the relationship between noise and CVD through other pathways. Therefore, we fitted a series of successive models: Model 1 adjusted for age and time period (by design); Model 2 further adjusted for a parsimonious set of covariates (race/ethnicity, marital status, spouse’s educational attainment, nSES score, ambient air pollution (PM_2.5_), population density, and region of residence); and Model 3 further adjusted for an extended set of covariates, adding individual-level health and health-related behavioral factors (physical activity, smoking status, smoking history in pack-years, diet, menopausal status, family history of MI). We created indicator variables for missing covariate data and included these in the models. In sensitivity analyses, we examined relationships between noise and our outcomes (incident CVD and all-cause mortality) using multiple imputation for missing covariate data (SAS procedure PROC MI/PROC MIANALYZE, SAS Institute, Cary, NC).

Analyses were conducted for NHS and NHSII separately as well as combined via DerSimonian and Laird estimators for random effects and inverse-variance weighting.^50^ We calculated *P* values for the Q-statistic to determine heterogeneity among the cohorts.

Primary analyses examined relationships between time-varying noise and CVD outcome using DNL dichotomized at 50 dB(A), comparing noise levels ≥50 dB(A) with noise levels <50 dB(A), based on having a sufficient sample size in each exposure bin. We also examined exposure–response associations by categorizing noise estimates into four categories [<45 dB(A) (reference), 45–49 dB(A), 50–54 dB(A), ≥55 dB(A)] as well as continuously. As only 17% of the sample had aircraft noise estimates ≥45 dB(A), we included an indicator variable in the model for estimates <45 dB(A). For continuous estimates, we fit cubic splines and used likelihood ratio tests to assess potential deviations from linearity.^51^

We conducted sensitivity analyses in which we examined an alternative definition of DNL (<45 vs. ≥45 dB(A)) and a broader definition of all-cause mortality (including both accidental and nonaccidental deaths, given relationships between noise and sleep). We also conducted sensitivity analyses restricting to individuals living in areas with modeled aircraft noise measurements below the FAA threshold for noise abatement (<65 dB(A)) as a proxy to exclude those who may have had abatement done in their homes.

We used SAS 9.4 (SAS Institute) for all analyses.

## Results

The overall sample consisted of 117,364 participants who contributed 1,706,278 person-years; 57,306 women contributed 814,128 person-years from NHS and 60,058 women contributed 892,150 person-years from NHSII (Table 1). Sample sizes within the all-cause mortality cohort were similar (Supplemental Table 1; http://links.lww.com/EE/A228). Participants in the study were mostly women who identified as non-Hispanic White and/or did not smoke. NHS participants were older and more likely to be postmenopausal, smoke, and be less physically active than NHSII participants. When examining baseline characteristics by aircraft noise exposure status, a larger percent of participants who were exposed to higher levels of aircraft noise lived in the Northeast and in more densely populated areas. Additionally, those exposed to higher levels of aircraft noise were less likely to identify as non-Hispanic White in both cohorts. Among participants living within the 22.2-mile radius airport buffers, about 16.5% of NHS participants and 17.5% of NHSII participants were located within modeled noise contours, with a range of noise exposure between 45 and 72 dB(A). Approximately 6.9% and 7.4% of NHS and NHSII participants, respectively, were exposed to DNL 50 dB(A) or higher (Supplemental Table 2 and Supplemental Figure 1; http://links.lww.com/EE/A228). Aircraft noise exposure was weakly correlated with population density, nSES score, and PM_2.5_ (Spearman r coefficients = −0.02 to 0.13).

**Table 1.** Age-standardized characteristics of NHS (1994) and NHSII (1995) participants at baseline who live near 90 major airports, overall and by dichotomized aircraft noise exposure.

		NHS			NHSII	
	Overall^a^	DNL < 50 dB(A)^a^	DNL ≥ 50 dB(A)^a^	Overall^a^	DNL < 50 dB(A)^a^	DNL ≥ 50 dB(A)^a^
N	57,306	53,483	3,823	60,058	55,675	4,383
Age, years^b^	61.1 ± 7.9	61.1 ± 7.9	61.2 ± 7.5	41.1 ± 5.6	41.2 ± 5.6	40.8 ± 5.4
Non-Hispanic White, %	90.8	91.1	86.2	89.3	89.8	83.2
Postmenopausal, %	88.1	88.3	86.5	9.6	9.7	8.8
Family history of MI, %	33.0	33.1	31.4	21.8	21.7	23.7
Married, %	60.0	60.4	54.7	61.0	61.4	55.7
Spouse’s highest level of education attainment, %						
Less than high school	3.4	3.4	4.0	0.5	0.5	0.6
High school	22.5	22.4	23.9	10.3	10.2	11.7
More than high school	37.5	37.9	31.6	64.9	65.2	60.8
Not married or missing	36.6	36.3	40.4	24.3	24.1	26.9
Smoking status, %						
Never smoker	42.2	42.2	41.8	63.2	63.4	60.5
Past smoker	41.4	41.5	40.1	25.3	25.3	25.8
Current smoker	12.5	12.4	13.4	10.4	10.3	12.1
Missing, %	3.9	3.9	4.6	1.1	1.1	1.6
Smoking history, pack-years	12.9 ± 19.7	12.8 ± 19.6	13.2 ± 20.1	4.7 ± 8.6	4.6 ± 8.6	5.2 ± 9.1
Missing, %	5.5	5.4	6.7	0.6	0.6	0.4
Alcohol consumption, %						
None	27.7	27.7	27.8	27.3	27.4	26.0
1 to <5 g/day	23.8	23.8	23.5	28.0	28.1	26.8
5 to <15 g/day	14.6	14.8	11.9	13.8	13.9	12.5
15 to <30 g/day	4.6	4.7	3.7	2.9	2.9	2.4
≥30 g/day	2.7	2.7	1.9	1.1	1.1	1.0
Missing, %	26.6	26.3	31.4	26.9	26.5	31.3
AHEI diet score	35.4 ± 23.1	35.6 ± 23.0	32.7 ± 23.8	33.4 ± 22.2	33.6 ± 22.1	31.5 ± 23.1
Missing, %	26.6	26.3	31.4	26.9	26.5	31.3
Physical activity, %						
<3 MET-hours/week	15.2	15.1	16.2	13.2	13.1	13.8
3-8 MET-hours/week	18.0	18.0	18.1	20.0	20.1	18.9
9–17 MET-hours/week	16.7	16.7	16.0	19.0	19.0	18.4
18–26 MET-hours/week	10.7	10.7	9.1	11.8	11.9	11.0
≥27 MET-hours/week	18.3	18.6	14.7	22.2	22.3	21.8
Missing, %	21.2	20.9	25.9	13.8	13.6	16.2
nSES score	−1.1 ± 2.8	−1.1 ± 2.8	−1.2 ± 2.5	−1.3 ± 2.7	−1.3 ± 2.7	−1.4 ± 2.7
PM_2.5_, µg/m³	13.9 ± 3.0	13.9 ± 3.0	14.6 ± 2.9	14.7 ± 3.3	14.7 ± 3.3	15.1 ± 3.3
Missing, %	0.2	0.2	0.3	0.3	0.3	0.4
Population density, persons/km^2^	1,935 ± 3,783	1,825 ± 3,684	3,523 ± 4,712	2,156 ± 5,131	2,048 ± 5,079	3,587 ± 5,574
Missing, %	11.3	11.2	14.1	9.6	9.4	12.5
Region of residence, %						
Northeast	44.2	43.7	51.5	33.1	32.3	42.6
Midwest	13.2	13.4	10.4	26.7	27.1	21.1
South	15.8	16.1	11.9	18.5	18.9	13.9
West	19.4	19.6	16.8	21.6	21.5	22.3
Missing, %	7.3	7.3	9.5	0.2	0.2	0.1

^a^Values are means ± standard deviations (SD) for continuous variables; percentages for categorical variables and are standardized to the age distribution of the study population.

^b^Value is not age adjusted.

AHEI indicates alternate healthy eating index.

Over the course of follow-up, 3915 participants from NHS and 614 participants from NHSII reported CVD (4529 total CVD cases). In combined analyses, when examining relationships between DNL dichotomized at 50 dB(A) and CVD, we found an HR of 1.00 (95% CI: 0.89, 1.12) adjusting for confounders in the parsimonious model, and an HR of 0.97 (95% CI: 0.86, 1.09) in the extended model (Table 2). Similar results were found in cohort-specific models. Effect estimates for relationships with continuous and alternate categorical noise metrics with CVD were also similar, with no indication of an exposure–response pattern (Table 2 and Supplemental Table 3; http://links.lww.com/EE/A228). Deviations from linearity were not observed for continuous models (*P* = 0.40 to 0.98; Supplemental Figure 2; http://links.lww.com/EE/A228), nor did we observe heterogeneity in the estimates from the two cohorts (*P* = 0.61 to 0.93). Models using multiple imputation methods yielded similar results to those with the indicator method (Supplemental Table 7; http://links.lww.com/EE/A228).

**Table 2.** Hazard ratios (95% confidence intervals) for associations between aircraft noise exposure (DNL) and CVD incidence in NHS and NHSII participants living near 90 major airports.

DNL, dB(A)	Cases	Person-years	Basic^a^	Parsimonious^b^	Extended^c^
NHS					
2-Category					
≥50	273	56,126	1.01 (0.89, 1.14)	1.00 (0.88, 1.14)	0.98 (0.86, 1.11)
<50	3,642	758,002	Ref	Ref	Ref
4-Category					
≥55	91	18,208	0.99 (0.80, 1.23)	0.98 (0.79, 1.21)	0.96 (0.77, 1.19)
50–54	182	37,918	1.02 (0.87, 1.18)	1.01 (0.87, 1.18)	0.98 (0.85, 1.15)
45–49	367	78,549	0.98 (0.88, 1.10)	0.98 (0.88, 1.10)	0.98 (0.88, 1.09)
<45	3,275	679,452	Ref	Ref	Ref
Continuous per 10 dB(A)	3,915	814,128	0.99 (0.82, 1.18)	0.97 (0.81, 1.17)	0.96 (0.80, 1.15)
NHSII					
2-Category					
≥50	44	66,516	1.00 (0.73, 1.36)	0.97 (0.71, 1.33)	0.93 (0.68, 1.28)
<50	570	825,633	Ref	Ref	Ref
4-Category					
≥55	16	22,195	1.13 (0.68, 1.86)	1.10 (0.66, 1.81)	1.06 (0.64, 1.75)
50–54	28	44,321	0.95 (0.65, 1.39)	0.93 (0.63, 1.36)	0.89 (0.60, 1.30)
45–49	68	90,585	1.11 (0.86, 1.43)	1.07 (0.83, 1.39)	1.06 (0.82, 1.38)
<45	502	735,049	Ref	Ref	Ref
Continuous per 10 dB(A)	614	892,150	1.05 (0.68, 1.62)	1.05 (0.68, 1.63)	1.01 (0.65, 1.56)
Meta-analysis^d^					
2-Category					
≥50	317	122,642	1.01 (0.90, 1.13)	1.00 (0.89, 1.12)	0.97 (0.86, 1.09)
<50	4,212	1,583,635	Ref	Ref	Ref
4-Category					
≥55	107	40,403	1.01 (0.83, 1.23)	1.00 (0.82, 1.21)	0.97 (0.80, 1.18)
50–54	210	82,239	1.01 (0.88, 1.16)	1.00 (0.87, 1.15)	0.97 (0.84, 1.12)
45–49	435	169,134	1.00 (0.91, 1.11)	1.00 (0.90, 1.10)	0.99 (0.90, 1.10)
<45	3,777	1,414,501	Ref	Ref	Ref
Continuous per 10 dB(A)	4,529	1,706,278	0.99 (0.84, 1.18)	0.98 (0.83, 1.16)	0.97 (0.82, 1.15)

^a^Basic models are stratified by age and calendar year.

^b^Parsimonious models are stratified by age and time period and adjusted for race/ethnicity, marital status, spouse’s education attainment, nSES score, region of residence, PM_2.5_, and population density.

^c^Extended models are stratified by age and time period and adjusted for race/ethnicity, marital status, spouse’s education attainment, nSES score, region of residence, PM_2.5_, population density, physical activity, smoking status, alcohol use, AHEI diet score, menopausal status, and family history of MI.

^d^*P* values for heterogeneity between NHS and NHSII cohorts range from 0.40 to 0.93.

AHEI indicates alternate healthy eating index.

Regarding all-cause mortality, 13,774 deaths in NHS and 1156 deaths in NHSII occurred over follow-up (14,930 total deaths). In pooled analyses, we observed HRs of 1.02 (95% CI: 0.96, 1.09) and 0.98 (95% CI: 0.92, 1.04) in parsimonious and extended models, respectively (Table 3), with no observed heterogeneity between NHS and NHSII cohorts (*P* = 0.16 to 0.95). These associations were robust among NHS and NHSII cohorts as well as across categorical and continuous noise metrics, with no evidence of exposure–response relationships (Table 3 and Supplemental Table 4; http://links.lww.com/EE/A228) or evidence of nonlinearity in continuous noise models (*P* = 0.11 to 0.94; Supplemental Figure 2; http://links.lww.com/EE/A228). Models using multiple imputation did not yield substantially different results from models using the indicator method (Supplemental Table 8; http://links.lww.com/EE/A228).

**Table 3.** Hazard ratios (95% confidence intervals) for associations between aircraft noise exposure (DNL) and all-cause mortality in NHS and NHSII participants living near 90 major airports.

DNL, dB(A)	Cases	Person-years	Basic^a^	Parsimonious^b^	Extended^c^
NHS					
2-Category					
≥50	976	58,394	1.08 (1.02, 1.16)	1.02 (0.95, 1.09)	0.98 (0.91, 1.05)
<50	12,798	790,557	Ref	Ref	Ref
4-Category					
≥55	335	18,950	1.15 (1.03, 1.28)	1.04 (0.93, 1.17)	1.02 (0.91, 1.14)
50–54	641	39,443	1.06 (0.98, 1.15)	1.00 (0.93, 1.09)	0.95 (0.88, 1.04)
45–49	1,290	81,962	1.03 (0.97, 1.09)	0.99 (0.93, 1.05)	0.99 (0.93, 1.05)
<45	11,508	708,595	Ref	Ref	Ref
Continuous per 10 dB(A)	13,774	848,950	1.08 (0.98, 1.19)	1.02 (0.93, 1.13)	1.00 (0.91, 1.10)
NHSII					
2-Category					
≥50	93	66,908	1.15 (0.93, 1.42)	1.04 (0.84, 1.29)	1.01 (0.81, 1.25)
<50	1,063	830,472	Ref	Ref	Ref
4-Category					
≥55	25	22,346	0.93 (0.62, 1.38)	0.79 (0.53, 1.18)	0.76 (0.51, 1.13)
50–54	68	44,562	1.25 (0.98, 1.60)	1.15 (0.90, 1.48)	1.12 (0.87, 1.44)
45–49	107	91,184	0.95 (0.78, 1.16)	0.89 (0.72, 1.09)	0.90 (0.73, 1.10)
<45	956	739,288	Ref	Ref	Ref
Continuous per 10 dB(A)	1,156	897,380	1.14 (0.83, 1.56)	1.05 (0.77, 1.44)	1.01 (0.74, 1.39)
Meta-analysis^d^					
2-Category					
≥50	1,069	125,302	1.09 (1.02, 1.16)	1.02 (0.96, 1.09)	0.98 (0.92, 1.04)
<50	13,861	1,621,029	Ref	Ref	Ref
4-Category					
≥55	360	41,296	1.13 (1.00, 1.27)	0.97 (0.77, 1.23)	0.94 (0.72, 1.22)
50–54	709	84,005	1.10 (0.96, 1.27)	1.02 (0.94, 1.11)	0.99 (0.87, 1.13)
45–49	1,397	173,146	1.02 (0.96, 1.08)	0.98 (0.93, 1.04)	0.98 (0.93, 1.04)
<45	12,464	1,447,883	Ref	Ref	Ref
Continuous per 10 dB(A)	14,930	1,746,330	1.09 (0.99, 1.19)	1.03 (0.94, 1.12)	1.00 (0.91, 1.10)

^a^ Basic models are stratified by age and calendar year.

^b^Parsimonious models are stratified by age and time period and adjusted for race/ethnicity, marital status, spouse’s education attainment, nSES score, region of residence, PM_2.5_, and population density.

^c^Extended models are stratified by age and time period and adjusted for race/ethnicity, marital status, spouse’s education attainment, nSES score, region of residence, PM_2.5_, population density, physical activity, smoking status, alcohol use, AHEI diet score, menopausal status, and family history of MI.

^d^*P* values for heterogeneity between NHS and NHSII cohorts range from 0.16 to 0.93.

AHEI indicates alternate healthy eating index.

Given the limited number of CVD deaths among participants exposed to DNL ≥50 dB(A) in NHSII (n = 12), we were only able to examine relationships between aircraft noise and CVD mortality in NHS (Table 4). Effect estimates for this relationship with dichotomized noise were larger than for all-cause mortality, with HRs of 1.07 (95% CI: 0.94, 1.23) and 1.03 (95% CI: 0.90, 1.19) in parsimonious and fully adjusted models, respectively; however, given the smaller case numbers, CIs were wider.

**Table 4.** Hazard ratios (95% confidence intervals) for associations between aircraft noise exposure (DNL) and cardiovascular mortality in NHS.

DNL, dB(A)	Cases	Person-years	Basic^a^	Parsimonious^b^	Extended^c^
2-Category					
≥50	239	59,071	1.15 (1.01, 1.32)	1.07 (0.94, 1.23)	1.03 (0.90, 1.19)
<50	2,899	799,674	Ref	Ref	Ref
4-Category					
≥55	75	19,175	1.10 (0.88, 1.39)	1.00 (0.79, 1.27)	0.99 (0.78, 1.25)
50–54	164	39,896	1.18 (1.01, 1.39)	1.11 (0.94, 1.30)	1.06 (0.90, 1.24)
45–49	297	82,873	1.03 (0.91, 1.16)	0.99 (0.88, 1.12)	1.00 (0.88, 1.13)
<45	2,602	716,800	Ref	Ref	Ref
Continuous per 10 dB(A)	3,138	858,745	1.07 (0.88, 1.30)	0.98 (0.81, 1.20)	0.96 (0.78, 1.17)

^a^Basic models are stratified by age and calendar year.

^b^Parsimonious models are stratified by age and time period and adjusted for race/ethnicity, marital status, spouse’s education attainment, nSES score, region of residence, PM_2.5_, and population density.

^c^Extended models are stratified by age and time period and adjusted for race/ethnicity, marital status, spouse’s education attainment, nSES score, region of residence, PM_2.5_, population density, physical activity, smoking status, alcohol use, AHEI diet score, menopausal status, and family history of MI.

AHEI indicates alternate healthy eating index.

When broadening our definition of all-cause mortality to accidental and nonaccidental deaths, we found that results did not change (Supplemental Table 5; http://links.lww.com/EE/A228). In sensitivity analyses restricting analyses to individuals living in areas below the FAA threshold for noise abatement (<65 dB(A)), we found similar results to the main analyses (Supplemental Table 6; http://links.lww.com/EE/A228).

## Discussion

Overall, we did not find evidence of an association between long-term exposure to aircraft noise and incidence of CVD or mortality. When examining cardiovascular-specific mortality, limited sample size constrained our analyses to NHS. In this group there was suggestive evidence of a 3% increased hazard of CVD mortality in our extended model, though these estimates were statistically imprecise. Our results were robust to various sensitivity analyses.

Overall, our results are generally consistent with studies worldwide examining relationships between aircraft noise and CVD, as evidence has been mixed. Vienneau and colleagues analyzed relationships of various transportation noise sources with CVD mortality and CVD mortality subtypes in a Swiss cohort; associations between aircraft specific noise and overall CVD mortality yielded similar results to our study, with an HR of 1.003 (95% CI: 0.996, 1.010).^27^ In a case-control study examining aircraft noise and stroke in Germany, authors found odds ratios (ORs) ranging from 0.97 to 1.02 for different categories of aircraft noise exposures compared with <40 dB(A).^6^ The authors had previously conducted a similar analysis examining MI as their outcome and found somewhat comparable results; however, the association examining individuals exposed to aircraft noise ≥65 dB(A) compared with <40 dB(A) produced a large yet imprecise OR of 1.42 (95% CI: 0.62, 3.25).^5^ A cross-sectional study conducted in six European countries examining aircraft noise with CVD found ORs of 1.06 (95% CI: 0.92, 1.21) and 1.12 (95% CI: 0.98, 1.29) per 10 dB(A) for daytime and nighttime noise, respectively.^3^ These associations were stronger among those living in homes for at least 20 years.

The majority of noise studies involving CVD outcomes have focused on mortality from CVD, particularly CVD subtypes. This literature hints at different biological mechanisms at play related to aircraft noise exposure and mortality. In a Swiss National Cohort study of over four million adults, authors did not find associations between aircraft noise (DNL) and all-cause mortality (HR: 1.0; 95% CI: 0.96, 1.1) yet found suggestive associations between aircraft noise and MI-specific mortality (HR: 1.30, 95% CI: 0.96, 1.70).^19^ Similarly, Vienneau et al did not find associations between aircraft noise and overall CVD mortality, yet they did find positive associations between aircraft noise and MI, heart failure, and ischemic stroke related deaths.^27^ Héritier and colleagues observed an HR of 0.99 (95% CI: 0.99, 1.00) for CVD mortality per 10 dB(A) increase in noise estimates^52^; and in examining mortality according to sub-CVD categories, observed HRs for MI-specific mortality of 1.03 (95% CI: 1.01, 1.05) and for ischemic stroke mortality of 1.07 (95% CI: 1.02, 1.13).^52^ Given the relatively low increases in risk observed in previous studies, it is very likely that our study was underpowered to observe associations between aircraft noise and CVD, as we only had 317 incident CVD cases and 251 CVD mortality cases exposed at levels above 50 dB(A). Furthermore, as our study and previous studies have not seen associations with overall CVD mortality, it would be informative to distinguish CVD endpoints (if power allows), as the effects of aircraft noise may differ depending on cardiovascular etiology. In this study, we were unable to stratify into CVD endpoints given the low number of exposed cases (Supplemental Table 9; http://links.lww.com/EE/A228).

This study adds to the limited number of studies that have been conducted on aircraft noise exposure and cardiovascular disease in the United States. Correia and colleagues used Medicare data to examine relationships between DNL estimates from 89 US airports and CVD hospitalizations at the zip-code level.^18^ They observed a 3.5% (95% CI: 0.2, 7.0) higher rate of CVD-related hospitalizations for every 10 dB(A) increase in DNL after adjusting for area-level confounders. Although the dataset was large (n = 6,027,363), it was cross-sectional in design as it examined noise exposure and hospitalizations only occurring in 2009. Additionally, authors were unable to account for individual-level confounding factors, such as health behaviors or individual-level SES. A retrospective study conducted on a sample of 498 individuals undergoing clinical imaging at a Boston (US)-area hospital investigated relationships between combined aircraft and road traffic noise levels in 2014 and major adverse cardiovascular disease events (MACE) over a 5-year period.^25^ The study reported a HR of 1.34 (95% CI: 1.15, 1.57) for every 5-dB(A) increase in combined noise level.^25^ Additionally, increased levels of amygdalar metabolic activity and arterial inflammation accounted for 12%–26% of the association between transportation noise and MACE, indicative of a potential mediating pathway between noise and cardiovascular morbidity and mortality.^25^

In this study, we did not examine relationships with nighttime noise due to small numbers with these exposures [8 CVD cases in NHS exposed to ≥50 dB(A) at night, 4 CVD cases in NHSII exposed to ≥50 dB(A) at night]. It is possible that we would observe stronger relationships between CVD incidence and nighttime noise given that sleep disruption is a proposed mechanism linking noise exposure to CVD incidence.^53,54^ For example, the Hypertension and Environmental Noise near Airports (HYENA) study surrounding six major European airports found positive associations between nighttime average aircraft noise exposure and self-reported CVD outcomes after adjustment for air pollution.^3^ Other observational and experimental studies have also found relationships between nighttime noise and CVD morbidity and mortality,^9,55–57^ particularly through oxidative stress pathways,^57–60^ thus suggesting noise exposure during certain time windows of the day may be particularly influential on cardiovascular health.^54,61,62^

This study has several limitations. First, very few individuals in our study were exposed to levels that might be typical at residences in close proximity to airports, as the NHS and NHSII cohorts were not designed to study noise exposure. Although the range of estimated exposure to aircraft noise extended from <45 to 72 dB(A), only about 2%, 0.5%, and 0.1% of each cohort were exposed at >55, >60, and >65 dB(A), respectively. These small numbers affected the precision of our estimates. Further affecting precision, participants of NHSII, by virtue of their age, were at particularly low risk for cardiovascular events and death.

Also constraining our study’s capacity to detect adverse effects of aircraft noise on CVD outcomes were aspects of the exposure ascertainment. We were limited to examining noise exposure using annual estimates derived once every 5 years and carried forward until the next 5-year time point. Additionally, we were unable to quantify noise exposures below 45 dB(A) with greater specificity. Even with perfectly measured long-term exposure to average daily exposure to aircraft noise, it is possible that exposures during different periods exert more cardiovascular hazard. For example, nighttime aircraft noise (e.g., 10 pm–6 am) may be more hazardous than daytime noise, or the effects of exposure may be acute and transient.^55^ We also did not have information on penetration of outdoor noise indoors, including factors that could affect penetration such as housing type, air conditioning, or window opening.

We did not adjust for a number of other potential confounders, such as other sources of noise (which were not linked to these cohorts). Although we did not adjust for other potential sources of noise, we hypothesize confounding from these sources would tend to upwardly bias the estimated HRs. Given that many HRs were <1 or indicated small positive effects, it seems unlikely that confounding of this nature strongly influenced our results. Our analyses include adjustments for air pollution and population density, which likely capture by proxy some of the effects of road noise. Additionally, we were unable to examine associations by perceptions of noise or annoyance. We were unable to assess length of residence; however, participants did not move frequently. We did conduct a sensitivity analysis in which we restricted the sample to only individuals who did not move during the study and found that effect estimates did not change for CVD incidence (Supplemental Table 10; http://links.lww.com/EE/A228); however, statistical models would not converge for mortality outcomes as we had further limited number of exposed cases.

It is possible that differential selection could have biased our estimates downward from a true positive association (i.e., HR >1). The most plausible mechanism leading to a downward bias is that the probability that a participant is included in the analyses (e.g., by being event-free at baseline, or remaining alive and in the study over follow-up) was differentially lower among persons with both higher exposure and higher CVD risk. A small reduction in selection probability in such persons could tilt a true null or small positive association toward an inverse one. In support of this bias are the lower HRs for incident CVD among groups with typically higher mortality risks—the older cohort (NHS) and among persons in both cohorts with lower nSES. The influence of differential selection and other sources of systematic error on estimated cardiovascular effects of aircraft noise merit further investigation. Even if the true effects are small, they may pose a substantial public health burden, given the large number of people exposed worldwide.

Last, these results may not be generalizable to the entire US population, as we studied cohorts that were majority non-Hispanic White female nurses. A recent study examining noise trends in the United States found that although there was considerable variability by airport, as racially and ethnically marginalized communities were most exposed to aircraft noise.^33^

This study also has several strengths. This is one of the first US nationwide studies examining aircraft noise and CVD using prospective cohort data. We also had a wide range of detailed individual-level data over a span of 20 years and were able to link comprehensively, analogously modeled aircraft noise estimates. As such, we were able to adjust for factors and examine statistical interactions using the rich and comprehensive data collected from participants every 2 years.

## Conclusions

In this national study of women in the United States, we did not find associations between aircraft noise exposure and CVD incidence or mortality; however, our results should be interpreted with caution as our findings may reflect our limited statistical power. Future studies with potentially more relevant aircraft noise metrics, for example, exposures at night, and cohorts designed to study noise to address power limitations are warranted to confirm these results.

## Conflicts of interest statement

The authors declare that they have no conflicts of interest with regard to the content of this report.

## Supplementary Material
