# Supplementary material for: Associations between long-term aircraft noise exposure, cardiovascular disease, and mortality in US cohorts of female nurses
Source: Environ Epidemiol. 2023 Jun 21;7(4):e259. doi: 10.1097/EE9.0000000000000259 (PMC10402956; doi:10.1097/EE9.0000000000000259)
Supplement: Supplementary file 1 [file ee9-7-e259-s001.docx]

**Supplemental Figure 1. Distribution of DNL Exposure in NHS (A) and NHSII (B).
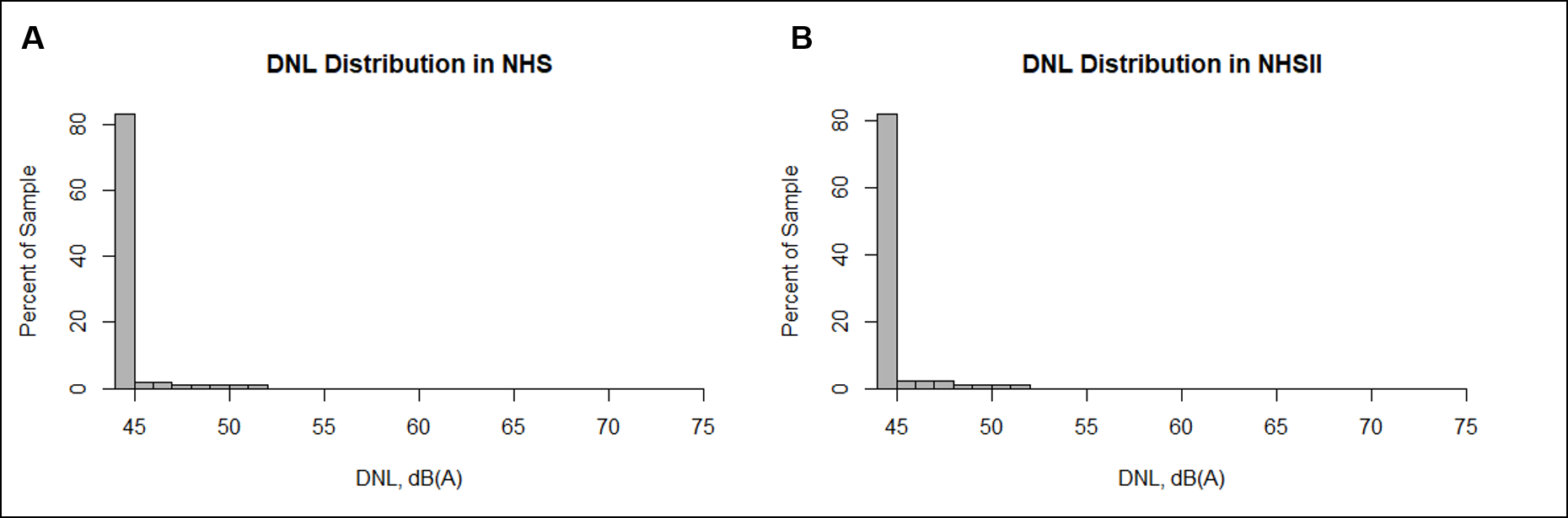
**

**Supplemental Figure 2. Hazard ratios (95% confidence intervals) for parsimonious^a^ associations between aircraft noise exposure (DNL) and CVD incidence (panels A-B) and all-cause mortality (panels C-D) in NHS and NHSII using cubic splines.**

**
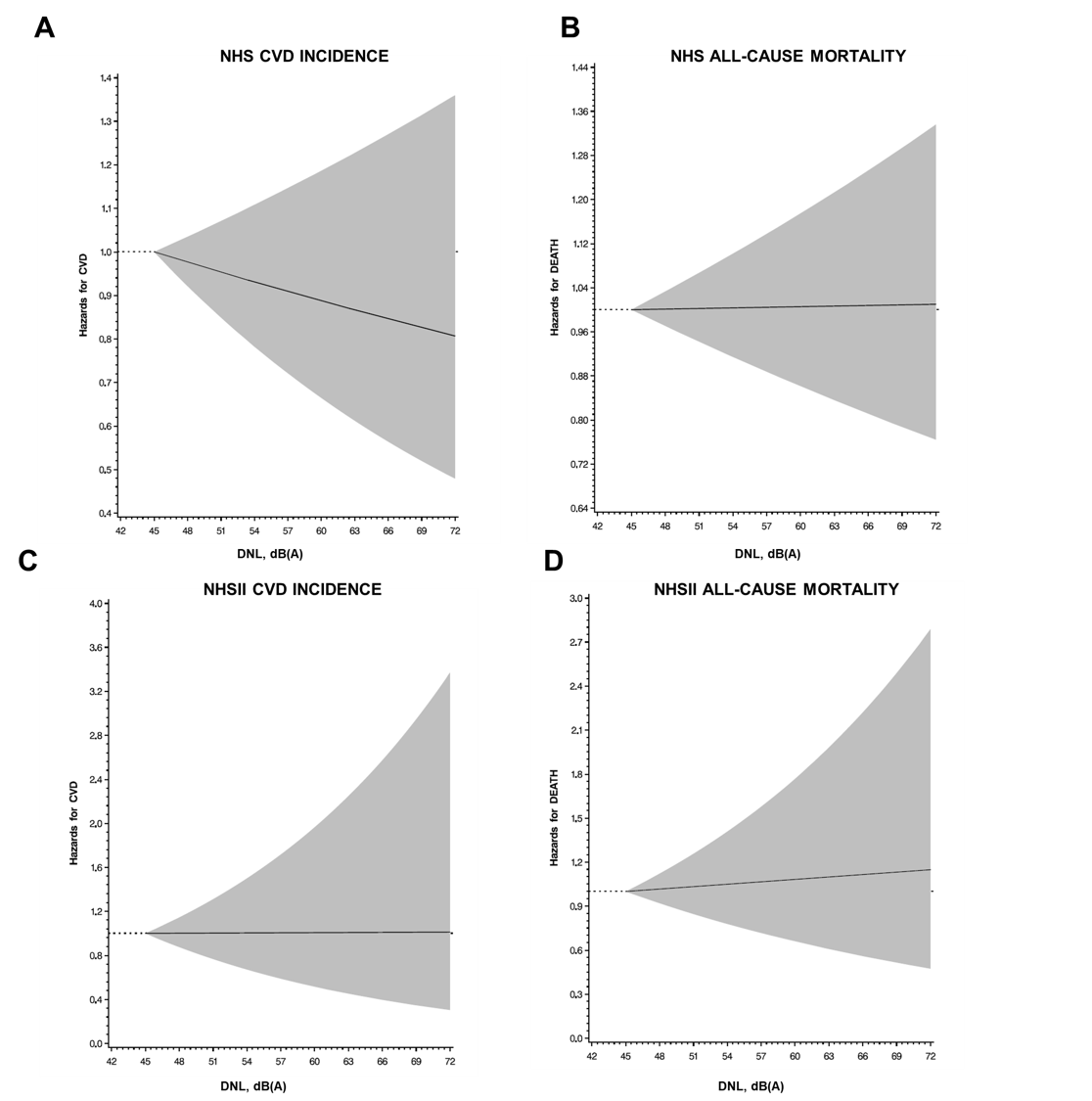
**

^a^ Parsimonious models are stratified by age and time period and adjusted for race/ethnicity, marital status, spouse’s education attainment, nSES score, region of residence, PM_2.5_, and population density.

Abbreviations: dB(A), A-weighted decibels; CVD, cardiovascular disease; DNL, day-night average sound level; NHS, Nurses' Health Study; NHSII, Nurses' Health Study II; nSES, neighborhood-level socioeconomic status; PM_2.5_, fine particulate matter.

**Supplemental Table 1. Age-standardized characteristics of Nurses’ Health Study (1994) and Nurses’ Health Study II (1995) participants at baseline who live near 90 major airports, overall and by aircraft noise exposure for all-cause mortality.**

|  |  | NHS |  |  | NHSII |  |
| --- | --- | --- | --- | --- | --- | --- |
|  | Overall ^a^ | DNL <50 dB(A) ^a^ | DNL ≥50 dB(A) ^a^ | Overall ^a^ | DNL <50 dB(A) ^a^ | DNL ≥50 dB(A) ^a^ |
| N | 58,710 | 54,794 | 3,916 | 60,174 | 55,781 | 4,393 |
| Age, yrs ^b^ | 61.3 ± 8.0 | 61.3 ± 8.0 | 61.3 ± 7.6 | 41.1 ± 5.6 | 41.2 ± 5.6 | 40.8 ± 5.4 |
| White Non-Hispanic, % | 90.8 | 91.1 | 86.4 | 89.3 | 89.7 | 83.2 |
| Post-Menopausal, % | 88.4 | 88.5 | 86.8 | 9.6 | 9.7 | 8.9 |
| Family History of MI, % | 33.2 | 33.3 | 31.8 | 21.9 | 21.7 | 23.8 |
| Married, % | 59.8 | 60.1 | 54.7 | 61.0 | 61.4 | 55.7 |
| Spouse's Highest Level of Education Attainment, % |  |  |  |  |  |  |
| Less than High School | 3.5 | 3.4 | 4.1 | 0.5 | 0.5 | 0.6 |
| High School | 22.6 | 22.5 | 24.2 | 10.3 | 10.2 | 11.7 |
| More than High School | 37.3 | 37.8 | 31.5 | 64.9 | 65.2 | 60.8 |
| Not Married or Missing | 36.6 | 36.3 | 40.2 | 24.3 | 24.1 | 26.9 |
| Smoking Status, % |  |  |  |  |  |  |
| Never Smoker | 41.9 | 41.9 | 41.3 | 63.1 | 63.3 | 60.4 |
| Past Smoker | 41.7 | 41.8 | 40.7 | 25.3 | 25.3 | 25.9 |
| Current Smoker | 12.5 | 12.5 | 13.5 | 10.4 | 10.3 | 12.1 |
| Missing, % | 3.9 | 3.8 | 4.6 | 1.1 | 1.1 | 1.6 |
| Smoking History, pack-yrs | 13.1 ± 20.0 | 13.1 ± 19.9 | 13.6 ± 20.5 | 4.7 ± 8.6 | 4.7 ± 8.6 | 5.2 ± 9.1 |
| Missing, % | 5.4 | 5.3 | 6.6 | 0.6 | 0.6 | 0.4 |
| Alcohol Consumption, % |  |  |  |  |  |  |
| None | 27.9 | 27.9 | 28.0 | 27.3 | 27.5 | 26.0 |
| 1 to <5 g/day | 23.6 | 23.6 | 23.4 | 28.0 | 28.1 | 26.8 |
| 5 to <15 g/day | 14.5 | 14.7 | 11.9 | 13.8 | 13.9 | 12.5 |
| 15 to <30 g/day | 4.6 | 4.6 | 3.6 | 2.9 | 2.9 | 2.4 |
| ≥30 g/day | 2.7 | 2.7 | 1.9 | 1.1 | 1.1 | 1.0 |
| Missing, % | 26.7 | 26.4 | 31.2 | 26.9 | 26.5 | 31.3 |
| AHEI Diet Score | 35.4 ± 23.1 | 35.6 ± 23.1 | 32.8 ± 23.8 | 33.4 ± 22.2 | 33.6 ± 22.1 | 31.6 ± 23.1 |
| Missing, % | 26.7 | 26.4 | 31.2 | 26.9 | 26.5 | 31.3 |
| Physical Activity, % |  |  |  |  |  |  |
| <3 MET-hrs/wk | 15.4 | 15.3 | 16.4 | 13.2 | 13.1 | 13.8 |
| 3-8 MET-hrs/wk | 18.1 | 18.1 | 18.3 | 20.0 | 20.1 | 18.9 |
| 9-17 MET-hrs/wk | 16.5 | 16.6 | 15.9 | 19.0 | 19.0 | 18.4 |
| 18-26 MET-hrs/wk | 10.6 | 10.7 | 9.0 | 11.8 | 11.8 | 11.0 |
| ≥27 MET-hrs/wk | 18.2 | 18.4 | 14.6 | 22.2 | 22.3 | 21.8 |
| Missing, % | 21.3 | 21.0 | 25.7 | 13.8 | 13.6 | 16.2 |
| nSES Score | -1.1 ± 2.8 | -1.1 ± 2.8 | -1.2 ± 2.5 | -1.3 ± 2.7 | -1.3 ± 2.7 | -1.4 ± 2.7 |
| PM_2.5_, µg/m³ | 13.9 ± 3.0 | 13.9 ± 3.0 | 14.6 ± 2.9 | 14.7 ± 3.3 | 14.7 ± 3.3 | 15.1 ± 3.3 |
| Missing, % | 0.2 | 0.2 | 0.3 | 0.3 | 0.3 | 0.4 |
| Population Density, persons/km^2^ | 1,936 ± 3,777 | 1,827 ± 3,676 | 3,515 ± 4,725 | 2,158 ± 5,136 | 2,049 ± 5,085 | 3,585 ± 5,569 |
| Missing, % | 11.3 | 11.1 | 13.9 | 9.6 | 9.4 | 12.5 |
| Region of Residence, % |  |  |  |  |  |  |
| Northeast | 44.2 | 43.7 | 51.3 | 33.0 | 32.3 | 42.5 |
| Midwest | 13.2 | 13.4 | 10.3 | 26.7 | 27.1 | 21.1 |
| South | 15.8 | 16.1 | 12.1 | 18.5 | 18.9 | 13.9 |
| West | 19.4 | 19.6 | 16.9 | 21.6 | 21.5 | 22.3 |
| Missing, % | 7.3 | 7.2 | 9.5 | 0.2 | 0.2 | 0.1 |

^a^ Values are means ± standard deviations (SD) for continuous variables; percentages for categorical variables and are standardized to the age distribution of the study population.

^b^ Value is not age adjusted.

Abbreviations: AHEI, Alternate Healthy Eating Index; dB(A), A-weighted decibels; DNL, day-night average sound level; g/day, grams per day; MI, myocardial infarction; µg/m³, micrograms per cubic meter, NHS, Nurses' Health Study; NHSII, Nurses' Health Study II; nSES, neighborhood socioeconomic status; MET-hrs/wk, metabolic equivalent hours per week; PM_2.5_, fine particulate matter; persons/km^2^, persons per square kilometer; yrs, years.

**Supplemental Table 2. Distribution of aircraft noise exposure across NHS and NHSII participants at baseline (NHS: 1994, NHSII: 1995).**

| **DNL, dB(A)** | **Participants in CVD Sample** | | **Participants in All-Cause Mortality Sample** | |
| --- | --- | --- | --- | --- |
|  | **NHS**  **(n=57,306)** | **NHSII**  **(n=60,058)** | **NHS**  **(n=58,710)** | **NHSII**  **(n=60,174)** |
| 70-75 | 3 | 1 | 4 | 1 |
| 65-69 | 49 | 69 | 52 | 69 |
| 60-64 | 241 | 290 | 248 | 292 |
| 55-59 | 965 | 1,139 | 982 | 1,141 |
| 50-54 | 2,565 | 2,884 | 2,630 | 2,890 |
| 45-49 | 5,507 | 6,152 | 5,631 | 6,161 |
| <45 | 47,976 | 49,523 | 49,163 | 49,620 |

**Supplemental Table 3. Hazard ratios (95% confidence intervals) for associations between aircraft noise exposure (DNL) dichotomized at the 45 dB(A) cut-point and CVD incidence in NHS and NHSII.**

| DNL, dB(A) | Cases | Person-Years | Basic ^a^ | Parsimonious ^b^ | Extended ^c^ |
| --- | --- | --- | --- | --- | --- |
|  |  |  |  |  |  |
| **NHS** |  |  |  |  |  |
| *≥45* | 640 | 134,676 | 0.99 (0.91, 1.08) | 0.99 (0.91, 1.08) | 0.98 (0.90, 1.07) |
| *<45* | 3,275 | 679,452 | *Ref* | *Ref* | *Ref* |
|  |  |  |  |  |  |
| **NHSII** |  |  |  |  |  |
| *≥45* | 112 | 157,101 | 1.07 (0.87, 1.31) | 1.03 (0.84, 1.28) | 1.01 (0.82, 1.25) |
| *<45* | 502 | 735,049 | *Ref* | *Ref* | *Ref* |
| **Meta-Analysis**^d^ |  |  |  |  |  |
| *≥45* | 752 | 291,777 | 1.00 (0.93, 1.09) | 1.00 (0.92, 1.08) | 0.98 (0.91, 1.07) |
| *<45* | 3,777 | 1,414,501 | *Ref* | *Ref* | *Ref* |
|  |  |  |  |  |  |

^a^ Basic models are stratified by age and calendar year.

^b^ Parsimonious models are stratified by age and time period and adjusted for race/ethnicity, marital status, spouse’s education attainment, nSES score, region of residence, PM_2.5_, and population density.

^c^ Extended models are stratified by age and time period and adjusted for race/ethnicity, marital status, spouse’s education attainment, nSES score, region of residence, PM_2.5_, population density, physical activity, smoking status, alcohol use, AHEI diet score, menopausal status, and family history of MI.

^d^ p-values for heterogeneity between NHS and NHSII cohorts range from 0.54 to 0.76.

Abbreviations: AHEI, Alternate Healthy Eating Index; dB(A), A-weighted decibels; CVD, cardiovascular disease; DNL, day-night average sound level; NHS, Nurses' Health Study; NHSII, Nurses' Health Study II; nSES, neighborhood-level socioeconomic status; MI, myocardial infarction; PM_2.5_, fine particulate matter; Ref, reference.

**Supplemental Table 4. Hazard ratios (95% confidence intervals) for associations between aircraft noise exposure (DNL) dichotomized at the 45 dB(A) cut-point and all-cause mortality in NHS and NHSII.**

| DNL, dB(A) | Cases | Person-Years | Basic ^a^ | Parsimonious ^b^ | Extended ^c^ |
| --- | --- | --- | --- | --- | --- |
|  |  |  |  |  |  |
| **NHS** |  |  |  |  |  |
| *≥45* | 2,266 | 140,355 | 1.05 (1.00, 1.10) | 1.00 (0.95, 1.05) | 0.98 (0.94,1.03) |
| *<45* | 11,508 | 708,595 | *Ref* | *Ref* | *Ref* |
|  |  |  |  |  |  |
| **NHSII** |  |  |  |  |  |
| *≥45* | 200 | 158,092 | 1.03 (0.88, 1.20) | 0.95 (0.81, 1.11) | 0.94 (0.80, 1.10) |
| *<45* | 956 | 739,288 | *Ref* | *Ref* | *Ref* |
| **Meta-Analysis**^d^ |  |  |  |  |  |
| *≥45* | 2,466 | 298,447 | 1.05 (1.01, 1.10) | 1.00 (0.95, 1.04) | 0.98 (0.94, 1.03) |
| *<45* | 12,464 | 1,447,883 | *Ref* | *Ref* | *Ref* |
|  |  |  |  |  |  |

^a^ Basic models are stratified by age and calendar year.

^b^ Parsimonious models are stratified by age and time period and adjusted for race/ethnicity, marital status, spouse’s education attainment, nSES score, region of residence, PM_2.5_, and population density.

^c^ Extended models are stratified by age and time period and adjusted for race/ethnicity, marital status, spouse’s education attainment, nSES score, region of residence, PM_2.5_, population density, physical activity, smoking status, alcohol use, AHEI diet score, menopausal status, and family history of MI.

^d^ p-values for heterogeneity between NHS and NHSII cohorts range from 0.51 to 0.80.

Abbreviations: AHEI, Alternate Healthy Eating Index; dB(A), A-weighted decibels; CVD, cardiovascular disease; DNL, day-night average sound level; NHS, Nurses' Health Study; NHSII, Nurses' Health Study II; nSES, neighborhood-level socioeconomic status; MI, myocardial infarction; PM_2.5_, fine particulate matter; Ref, reference.

**Supplemental Table 5. Hazard ratios (95% confidence intervals) for associations between aircraft noise exposure (DNL) and all-cause mortality (defined as accidental and non-accidental deaths) in NHS and NHSII.**

| DNL, dB(A) | Cases | Person-Years | Basic ^a^ | Parsimonious ^b^ | Extended ^c^ |
| --- | --- | --- | --- | --- | --- |
|  |  |  |  |  |  |
| **NHS** |  |  |  |  |  |
| *2-Category* |  |  |  |  |  |
| *≥50* | 1,000 | 58,366 | 1.09 (1.02, 1.16) | 1.02 (0.96, 1.09) | 0.98 (0.92, 1.05) |
| *<50* | 13,090 | 790,301 | *Ref* | *Ref* | *Ref* |
|  |  |  |  |  |  |
| *4-Category* |  |  |  |  |  |
| *≥55* | 344 | 18,941 | 1.15 (1.04, 1.29) | 1.06 (0.95, 1.18) | 1.03 (0.93, 1.16) |
| *50-54* | 656 | 39,425 | 1.06 (0.98, 1.15) | 1.01 (0.93, 1.09) | 0.96 (0.88, 1.04) |
| *45-49* | 1,330 | 81,923 | 1.04 (0.98, 1.10) | 1.00 (0.94, 1.06) | 1.00 (0.94, 1.06) |
| *<45* | 11,760 | 708,377 | Ref | Ref | Ref |
|  |  |  |  |  |  |
| *Continuous per 10 dB(A)* | 14,090 | 848,667 | 1.07 (0.98, 1.18) | 1.02 (0.93, 1.12) | 1.00 (0.91, 1.10) |
|  |  |  |  |  |  |
|  |  |  |  |  |  |
| **NHSII** |  |  |  |  |  |
| *2-Category* |  |  |  |  |  |
| *≥50* | 104 | 66,898 | 1.14 (0.93, 1.39) | 1.04 (0.85, 1.28) | 1.01 (0.82, 1.24) |
| *<50* | 1,188 | 830,351 | *Ref* | *Ref* | *Ref* |
|  |  |  |  |  |  |
| *4-Category* |  |  |  |  |  |
| *≥55* | 30 | 22,344 | 0.99 (0.68, 1.42) | 0.85 (0.59, 1.23) | 0.82 (0.57, 1.19) |
| *50-54* | 74 | 44,554 | 1.21 (0.96, 1.54) | 1.13 (0.89, 1.44) | 1.10 (0.87, 1.40) |
| *45-49* | 124 | 91,169 | 0.98 (0.82, 1.18) | 0.93 (0.77, 1.13) | 0.94 (0.78, 1.14) |
| *<45* | 1,064 | 739,182 | *Ref* | *Ref* | *Ref* |
|  |  |  |  |  |  |
| *Continuous per 10 dB(A)* | 1,292 | 897,249 | 1.18 (0.88, 1.58) | 1.09 (0.82, 1.46) | 1.05 (0.79, 1.41) |
|  |  |  |  |  |  |
| **Meta-Analysis**^d^ |  |  |  |  |  |
| *2-Category* |  |  |  |  |  |
| *≥50* | 1,104 | 125,264 | 1.09 (1.03, 1.16) | 1.03 (0.96, 1.09) | 0.99 (0.92, 1.05) |
| *<50* | 14,278 | 1,620,652 | *Ref* | *Ref* | *Ref* |
|  |  |  |  |  |  |
| *4-Category* |  |  |  |  |  |
| *≥55* | 374 | 41,285 | 1.14 (1.03, 1.26) | 1.02 (0.88, 1.19) | 0.99 (0.83, 1.18) |
| *50-54* | 730 | 83,979 | 1.08 (0.99, 1.18) | 1.02 (0.94, 1.10) | 0.98 (0.88, 1.09 |
| *45-49* | 1,454 | 173,092 | 1.03 (0.98, 1.09) | 0.99 (0.94, 1.05) | 0.99 (0.94, 1.05 |
| *<45* | 12,824 | 1,447,559 | *Ref* | *Ref* | *Ref* |
|  |  |  |  |  |  |
| *Continuous per 10 dB(A)* | 15,382 | 1,745,916 | 1.08 (0.99, 1.18) | 1.03 (0.94, 1.12 | 1.00 (0.92, 1.10) |
|  |  |  |  |  |  |

^a^ Basic models are stratified by age and calendar year.

^b^ Parsimonious models are stratified by age and time period and adjusted for race/ethnicity, marital status, spouse’s education attainment, nSES score, region of residence, PM_2.5_, and population density.

^c^ Extended models are stratified by age and time period and adjusted for race/ethnicity, marital status, spouse’s education attainment, nSES score, region of residence, PM_2.5_, population density, physical activity, smoking status, alcohol use, AHEI diet score, menopausal status, and family history of MI.

^d^ p-value for heterogeneity between NHS and NHSII cohorts range from 0.24 to 0.86.

Abbreviations: AHEI, Alternate Healthy Eating Index; dB(A), A-weighted decibels; DNL, day-night average sound level; NHS, Nurses' Health Study; NHSII, Nurses' Health Study II; nSES, neighborhood-level socioeconomic status; MI, myocardial infarction; PM_2.5_, fine particulate matter; Ref, reference.

**Supplemental Table 6. Sensitivity analyses examining associations between DNL and incident CVD and all-cause mortality in NHS and NHSII when restricting to noise estimates below the FAA noise abatement threshold (<65 dB(A)).**

|  |  | *NHS* |  |  | *NHSII* |  |
| --- | --- | --- | --- | --- | --- | --- |
|  | Cases | Person-Years | Hazard Ratio (95% CI) | Cases | Person-Years | Hazard Ratio (95% CI) |
|  |  |  |  |  |  |  |
| *Incident CVD* |  |  |  |  |  |  |
| ≥50 dB(A) | 271 | 55,353 | 1.01 (0.89, 1.14) | 44 | 65,547 | 0.99 (0.72, 1.35) |
| <50 dB(A) | 3,642 | 758,002 | *Ref* | 570 | 825,633 | *Ref* |
|  |  |  |  |  |  |  |
| *All-Cause Mortality* |  |  |  |  |  |  |
| ≥50 dB(A) | 964 | 57,593 | 1.02 (0.96, 1.10) | 92 | 65,938 | 1.05 (0.84, 1.30) |
| <50 dB(A) | 12,798 | 790,557 | *Ref* | 1,063 | 830,472 | *Ref* |

Models are stratified by age and time period and adjusted for race/ethnicity, marital status, spouse’s education attainment, nSES score, region of residence, PM_2.5_, and population density.

Abbreviations: dB(A), A-weighted decibels; CI, confidence interval; CVD, cardiovascular disease; DNL, day-night average sound level; NHS, Nurses' Health Study; NHSII, Nurses' Health Study II; nSES, neighborhood-level socioeconomic status; PM_2.5_, fine particulate matter.

**Supplemental Table 7. Hazard ratios (95% confidence intervals) for associations between aircraft noise exposure (DNL) and incident CVD in NHS and NHSII using multiple imputation to address missing covariate information.**

| DNL, dB(A) | Parsimonious ^a^ | Extended ^b^ |
| --- | --- | --- |
|  |  |  |
| **NHS** |  |  |
| *2-Category* |  |  |
| *≥50* | 1.00 (0.88, 1.13) | 0.98 (0.86, 1.11) |
| *<50* | *Ref* | *Ref* |
|  |  |  |
| *4-Category* |  |  |
| *≥55* | 0.98 (0.79, 1.21) | 0.96 (0.78, 1.19) |
| *50-54* | 1.01 (0.87, 1.17) | 0.98 (0.85, 1.14) |
| *45-49* | 0.98 (0.87, 1.09) | 0.97 (0.87, 1.08) |
| *<45* | Ref | Ref |
|  |  |  |
| *Continuous per 10 dB(A)* | 0.98 (0.82, 1.17) | 0.97 (0.81, 1.16) |
|  |  |  |
|  |  |  |
| **NHSII** |  |  |
| *2-Category* |  |  |
| *≥50* | 0.97 (0.70, 1.34) | 0.93 (0.67, 1.29) |
| *<50* | *Ref* | *Ref* |
|  |  |  |
| *4-Category* |  |  |
| *≥55* | 1.14 (0.68, 1.92) | 1.09 (0.65, 1.84) |
| *50-54* | 0.90 (0.60, 1.35) | 0.87 (0.58, 1.31) |
| *45-49* | 1.09 (0.84, 1.42) | 1.09 (0.83, 1.42) |
| *<45* | *Ref* | *Ref* |
|  |  |  |
| *Continuous per 10 dB(A)* | 1.03 (0.65, 63) | 0.98 (0.62, 1.56) |
|  |  |  |
| **Meta-Analysis**^c^ |  |  |
| *2-Category* |  |  |
| *≥50* | 1.00 (0.89, 1.12) | 0.97 (0.87, 1.10) |
| *<50* | *Ref* | *Ref* |
|  |  |  |
| *4-Category* |  |  |
| *≥55* | 1.00 (0.82, 1.22) | 0.98 (0.80, 1.19) |
| *50-54* | 0.99 (0.86, 1.14) | 0.97 (0.84, 1.12) |
| *45-49* | 0.99 (0.90, 1.10) | 0.99 (0.89, 1.09) |
| *<45* | *Ref* | *Ref* |
|  |  |  |
| *Continuous per 10 dB(A)* | 0.99 (0.83, 1.17) | 0.97 (0.82, 1.15) |
|  |  |  |

^a^ Parsimonious models are stratified by age and time period and adjusted for race/ethnicity, marital status, spouse’s education attainment, nSES score, region of residence, PM_2.5_, and population density.

^b^ Extended models are stratified by age and time period and adjusted for race/ethnicity, marital status, spouse’s education attainment, nSES score, region of residence, PM_2.5_, population density, physical activity, smoking status, alcohol use, AHEI diet score, menopausal status, and family history of MI.

^c^ p-value for heterogeneity between NHS and NHSII cohorts range from 0.44 to 0.96.

Abbreviations: AHEI, Alternate Healthy Eating Index; dB(A), A-weighted decibels; DNL, day-night average sound level; NHS, Nurses' Health Study; NHSII, Nurses' Health Study II; nSES, neighborhood-level socioeconomic status; MI, myocardial infarction; PM_2.5_, fine particulate matter; Ref, reference.

**Supplemental Table 8. Hazard ratios (95% confidence intervals) for associations between aircraft noise exposure (DNL) and all-cause mortality in NHS and NHSII using multiple imputation to address missing covariate information.**

| DNL, dB(A) | Parsimonious ^a^ | Extended ^b^ |
| --- | --- | --- |
|  |  |  |
| **NHS** |  |  |
| *2-Category* |  |  |
| *≥50* | 1.06 (0.99, 1.13) | 1.02 (0.95, 1.09) |
| *<50* | *Ref* | *Ref* |
|  |  |  |
| *4-Category* |  |  |
| *≥55* | 1.10 (0.99, 1.23) | 1.07 (0.95, 1.19) |
| *50-54* | 1.03 (0.95, 1.12) | 1.00 (0.92, 1.08) |
| *45-49* | 1.01 (0.95, 1.07) | 1.00 (0.94, 1.06) |
| *<45* | Ref | Ref |
|  |  |  |
| *Continuous per 10 dB(A)* | 1.06 (0.97, 1.17) | 1.04 (0.95, 1.15) |
|  |  |  |
|  |  |  |
| **NHSII** |  |  |
| *2-Category* |  |  |
| *≥50* | 1.04 (0.84, 1.29) | 1.01 (0.82, 1.26) |
| *<50* | *Ref* | *Ref* |
|  |  |  |
| *4-Category* |  |  |
| *≥55* | 0.81 (0.54, 1.20) | 0.79 (0.53, 1.18) |
| *50-54* | 1.14 (0.89, 1.47) | 1.11 (0.86, 1.42) |
| *45-49* | 0.89 (0.73, 1.09) | 0.90 (0.73, 1.10) |
| *<45* | Ref | Ref |
|  |  |  |
| *Continuous per 10 dB(A)* | 1.06 (0.78, 1.46) | 1.04 (0.76, 1.43) |
|  |  |  |
| **Meta-Analysis**^c^ |  |  |
| *2-Category* |  |  |
| *≥50* | 1.05 (0.99, 1.12) | 1.02 (0.96, 1.09) |
| *<50* | *Ref* | *Ref* |
|  |  |  |
| *4-Category* |  |  |
| *≥55* | 1.00 (0.76, 1.33) | 0.98 (0.75, 1.28) |
| *50-54* | 1.04 (0.97, 1.13) | 1.01 (0.93, 1.09) |
| *45-49* | 0.99 (0.91, 1.08) | 0.99 (0.93, 1.06) |
| *<45* | *Ref* | *Ref* |
|  |  |  |
| *Continuous per 10 dB(A)* | 1.06 (0.97, 1.16) | 1.04 (0.95, 1.14) |
|  |  |  |

^a^ Parsimonious models are stratified by age and time period and adjusted for race/ethnicity, marital status, spouse’s education attainment, nSES score, region of residence, PM_2.5_, and population density.

^b^ Extended models are stratified by age and time period and adjusted for race/ethnicity, marital status, spouse’s education attainment, nSES score, region of residence, PM_2.5_, population density, physical activity, smoking status, alcohol use, AHEI diet score, menopausal status, and family history of MI.

^c^ p-value for heterogeneity between NHS and NHSII cohorts range from 0.14 to 0.99.

Abbreviations: AHEI, Alternate Healthy Eating Index; dB(A), A-weighted decibels; DNL, day-night average sound level; NHS, Nurses' Health Study; NHSII, Nurses' Health Study II; nSES, neighborhood-level socioeconomic status; MI, myocardial infarction; PM_2.5_, fine particulate matter; Ref, reference.

**Supplemental Table 9. Distribution of aircraft noise exposure across NHS and NHSII incident CHD cases.**

| **DNL, dB(A)** | **NHS ^a^** | | **NHSII ^b^** | |
| --- | --- | --- | --- | --- |
|  | CHD  (n=2,025) | Stroke  (n=1,923) | CHD  (n=315) | Stroke  (n=301) |
| ≥55 | 49 | 42 | 8 | 8 |
| 50-54 | 99 | 83 | 18 | 10 |
| 45-49 | 200 | 169 | 35 | 33 |
| <45 | 1,677 | 1,629 | 254 | 250 |

^a^ Thirty-three NHS participants had concurrent CHD and stroke events.

^b^ Two NHSII participants had concurrent CHD and stroke events.

Abbreviations: dB(A), A-weighted decibels; CHD, coronary heart disease; DNL, day-night average sound level; NHS, Nurses' Health Study; NHSII, Nurses' Health Study II.

**Supplemental Table 10. Hazard ratios (95% confidence intervals) for associations between aircraft noise exposure (DNL) and CVD incidence in NHS and NHSII participants living near 90 major airports, restricted to individuals who have not moved from their residence during the study.**

| DNL, dB | Cases | | Person-Years | | Basic ^a^ | | Parsimonious ^b^ | | Extended ^c^ |
| --- | --- | --- | --- | --- | --- | --- | --- | --- | --- |
|  |  | |  | |  | |  | |  |
| **NHS** |  | |  | |  | |  | |  |
| *2-Category* |  | |  | |  | |  | |  |
| *≥50* | 235 | | 48,348 | | 1.01 (0.88, 1.16) | | 1.00 (0.87, 1.14) | | 0.97 (0.84, 1.11) |
| *<50* | 2,877 | | 611,262 | | *Ref* | | *Ref* | | *Ref* |
|  |  | |  | |  | |  | |  |
| *4-Category* |  | |  | |  | |  | |  |
| *≥55* | 80 | | 15,750 | | 1.00 (0.80, 1.26) | | 0.98 (0.78, 1.23) | | 0.96 (0.76, 1.20) |
| *50-54* | 155 | | 32,598 | | 1.02 (0.86, 1.20) | | 1.01 (0.86, 1.19) | | 0.98 (0.83, 1.15) |
| *45-49* | 320 | | 67,068 | | 1.02 (0.90, 1.14) | | 1.02 (0.90, 1.14) | | 1.01 (0.89, 1.14) |
| *<45* | 2,557 | | 544,194 | | *Ref* | | *Ref* | | *Ref* |
|  |  | |  | |  | |  | |  |
| *Continuous per 10 dB* | 3,112 | | 659,610 | | 0.97 (0.80, 1.18) | | 0.96 (0.79, 1.17) | | 0.95 (0.78, 1.16) |
|  |  | |  | |  | |  | |  |
|  |  | |  | |  | |  | |  |
| **NHSII** |  | |  | |  | |  | |  |
| *2-Category* |  | |  | |  | |  | |  |
| *≥50* | 34 | | 46,007 | | 1.11 (0.78, 1.58) | | 1.08 (0.76, 1.55) | | 1.04 (0.72, 1.48) |
| *<50* | 367 | | 527,415 | | *Ref* | | *Ref* | | *Ref* |
|  |  | |  | |  | |  | |  |
| *4-Category* |  | |  | |  | |  | |  |
| *≥55* | 9 | | 15,709 | | 0.89 (0.46, 1.74) | | 0.86 (0.44, 1.68) | | 0.83 (0.42, 1.62) |
| *50-54* | 25 | | 30,298 | | 1.24 (0.83, 1.88) | | 1.21 (0.80, 1.83) | | 1.15 (0.76, 1.74) |
| *45-49* | 47 | | 61,725 | | 1.11 (0.81, 1.51) | | 1.07 (0.78, 1.46) | | 1.06 (0.78, 1.45) |
| *<45* | 320 | | 465,689 | | *Ref* | | *Ref* | | *Ref* |
|  |  | |  | |  | |  | |  |
| *Continuous per 10 dB* | 401 | | 573,422 | | 0.94 (0.56, 1.59) | | 0.95 (0.56, 1.59) | | 0.90 (0.53, 1.52) |
|  |  | |  | |  | |  | |  |
| **Meta-Analysis**^d^ | |  | |  | |  | |  | |
| *2-Category* |  | |  | |  | |  | |  |
| *≥50* | 269 | | 94,355 | | 1.02 (0.90, 1.16) | | 1.01 (0.89, 1.15) | | 0.98 (0.86, 1.11) |
| *<50* | 3,244 | | 1,138,677 | | *Ref* | | *Ref* | | *Ref* |
|  |  | |  | |  | |  | |  |
| *4-Category* |  | |  | |  | |  | |  |
| *≥55* | 89 | | 31,459 | | 0.99 (0.80, 1.23) | | 0.97 (0.78, 1.20) | | 0.94 (0.76, 1.17) |
| *50-54* | 180 | | 62,896 | | 1.04 (0.90, 1.22) | | 1.04 (0.89, 1.21) | | 1.00 (0.86, 1.17) |
| *45-49* | 367 | | 128,793 | | 1.03 (0.92, 1.15) | | 1.02 (0.91, 1.14) | | 1.01 (0.91, 1.13) |
| *<45* | 2,877 | | 1,009,883 | | *Ref* | | *Ref* | | *Ref* |
|  |  | |  | |  | |  | |  |
| *Continuous per 10 dB* | 3,513 | | 1,233,032 | | 0.97 (0.81, 1.16) | | 0.96 (0.80, 1.15) | | 0.94 (0.78, 1.14) |
|  |  | |  | |  | |  | |  |

^a^ Basic models are stratified by age and calendar year.

^b^ Parsimonious models are stratified by age and time period and adjusted for race/ethnicity, marital status, spouse’s education attainment, nSES score, region of residence, PM_2.5_, and population density.

^c^ Extended models are stratified by age and time period and adjusted for race/ethnicity, marital status, spouse’s education attainment, nSES score, region of residence, PM_2.5_, population density, physical activity, smoking status, alcohol use, AHEI diet score, menopausal status, and family history of MI.

^d^ p-values for heterogeneity between NHS and NHSII cohorts range from 0.37 to 0.95.

Abbreviations: AHEI, Alternate Healthy Eating Index; dB(A), A-weighted decibels; CVD, cardiovascular disease; DNL, day-night average sound level; NHS, Nurses' Health Study; NHSII, Nurses' Health Study II; nSES, neighborhood-level socioeconomic status; MI, myocardial infarction; PM_2.5_, fine particulate matter; Ref, reference.
